# Barriers and enablers to adolescent self-consent for vaccination: A mixed-methods evidence synthesis

**DOI:** 10.1016/j.vaccine.2018.12.007

**Published:** 2019-01-14

**Authors:** Harriet Fisher, Sarah Harding, Matthew Hickman, John Macleod, Suzanne Audrey

**Affiliations:** Population Health Sciences, Bristol Medical School, University of Bristol, United Kingdom

**Keywords:** Self-consent, Vaccination, Systematic review, Adolescents, Mixed methods, HPV, Human Papillomavirus, UK, United Kingdom, USA, United States of America, PRISMA, Preferred Reporting Items for Systematic Reviews and Meta-Analyses Protocol, PROSPERO, Prospective Register of Systematic Reviews, MeSH, Medical Subject Headings, OR, Odds Ratio

## Abstract

**Introduction:**

The recent global expansion of routine adolescent vaccination programmes has the potential to protect young people against infectious diseases and improve their health. Although the legal framework in many countries permits young people to consent for vaccinations if competent, lack of written parental consent can still prevent uptake. We aimed to review systematically the associated barriers and enablers to implementation of adolescent self-consent procedures.

**Methods:**

A comprehensive search strategy of ten databases from inception to June 2018 was undertaken to identify relevant qualitative and quantitative studies. Titles, abstracts and full texts were assessed for eligibility, and the methodological quality of eligible primary studies evaluated. Thematic synthesis methods were used to interpret and combine qualitative data, and to identify overarching themes as well as similarities and differences within themes. Quantitative data were summarised and, because the data were sufficiently similar in focus, were integrated within the qualitative framework.

**Results:**

Twenty-five publications related to 23 studies were included. Three themes were identified which related to the policy framework, protection, and self-determination. Despite supportive national policy frameworks, implementation of adolescent self-consent procedures can be prevented by local policies, professionals’ misunderstandings of the legal framework and the context in which the vaccination programme is delivered. Motivation to protect young people’s health increased acceptability of adolescent self-consent, but implementation might be prevented to protect the reputation of professionals or relationships with parents. Further, maintaining the role of parents as decision-makers for their child’s healthcare was frequently prioritised over enabling young people’s autonomy to consent.

**Conclusions:**

Barriers to the implementation of adolescent self-consent procedures have implications for young people’s health and uptake of vaccination programmes. There is a need to clarify the policy framework and challenge the primacy of parental consent.

**Systematic review registration:**

PROSPERO CRD42017084509.

## Highlights

• Synthesis of studies related to self-consent procedures for adolescent vaccination.
• Robust systematic review methodology used to identify and appraise the literature.
• There is a need to clarify policy and address professionals’ misunderstandings.
• Concerns about professional practice and relationships with parents create barriers.
• Enabling adolescent self-consent entails disputing the primacy of parental consent.

## 1 Introduction

The number of routine vaccinations recommended during adolescence is increasing, and includes vaccines that protect against acquisition of tetanus, diphtheria, meningococcal disease and human papillomavirus (HPV) [1], [2]. The expansion of adolescent vaccination programmes may improve young people’s health by protecting them from potentially life-threatening infectious diseases. In some countries, high uptake of adolescent vaccination programmes has been achieved [3], [4], [5]. However, national data often conceals within country inequalities in uptake and access. For example, in England the overall uptake of Td/IPV booster vaccination programme in 2016/17 was 82% (range: 44–100%). Further, in 14 of the 152 local authorities uptake was less than 70% [4]. Further, evidence from a systematic review and meta-analysis showed that young women from minority ethnic populations and without healthcare insurance were less likely to initiate the HPV vaccination series [6]. Targeting specific populations identified with lower uptake with focused interventions, such as introduction of adolescent self-consent procedures, could help ensure higher and more equitable uptake across all population groups.

The introduction of new adolescent vaccination programmes is relevant to the debate about young people’s capacity to provide consent to receive medical treatment. The United Nations Convention on the Rights of the Child recognises the right for all children and young people to participate in decision-making processes which involve them [7]. However, the World Health Organisation has acknowledged difficulties over consent for vaccination of adolescents because of their age, and describes current practice through which countries are encouraged to adopt procedures that ensure parents have been informed and have agreed to the vaccination [8].

In most countries, the legal framework for consent requires parental or guardian permission for young people aged below 18 years [8]. However, the age of consent for medical interventions, such as vaccination programmes, is lower in some countries. In the UK, Canada and Sweden, young women are legally able to override parental decisions if they are considered mature enough to make, and understand the consequences of, the decision to vaccinate. In Australia and the USA, there are geographic variations of the age (12–17 years) that a young person can consent to be vaccinated. Despite young people being supported by the law to provide consent themselves, written parental consent is usually sought.

It is currently unknown whether the introduction of adolescent self-consent procedures can increase uptake of adolescent vaccination programmes. This paper reports the findings of a mixed-methods systematic review aiming to identify, appraise and synthesise the available qualitative and quantitative literature relating to self-consent procedures for adolescent vaccination programmes. The original primary research question was to describe the effectiveness of self-consent interventions at increasing uptake of adolescent vaccination programmes. However, no such intervention studies were retrieved. As a result, the findings presented here are relevant to the secondary research objective: to report the barriers and enablers of implementation of self-consent procedures for adolescent vaccination programmes.

## 2 Material and methods

The full details of the methodology for this study are available in a published protocol [9]. This systematic review was registered with the International Prospective Register of Systematic Reviews (PROSPERO) (Registration number: CRD42017084509).

### 2.1 Search strategy and study selection

**Fig. 1:**
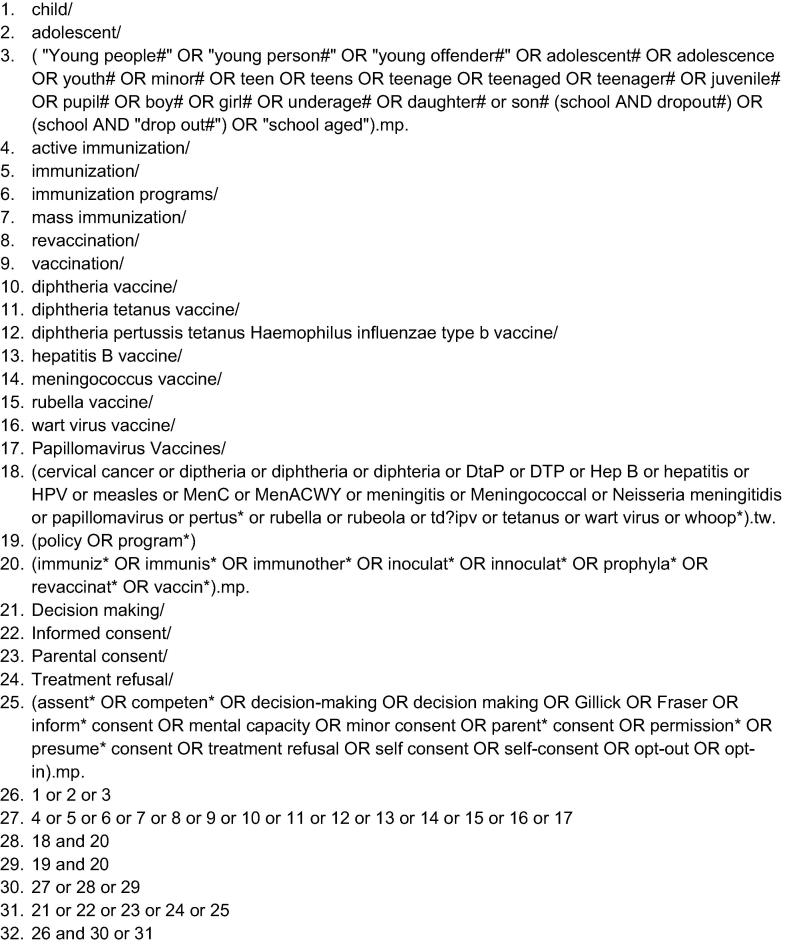
Embase search strategy.

A search strategy was developed which combined Medical Subject Headings (MeSH) for young people (e.g. child, adolescent), with terms for vaccination programmes (e.g. immunization) and self-consent (e.g. decision-making, informed consent) (Fig. 1). Studies for inclusion were identified through a broad search of ten databases proposed in the protocol [9] from inception to January 2018. The search was updated in June 2018.

Two reviewers (HF & SH) independently assessed the titles and abstracts, and full-text publications of all potentially relevant articles were retrieved and scrutinised for relevance. Disagreements were resolved by discussion. Reference lists and bibliographies from eligible studies and systematic reviews were hand-searched. The reference management software EndNote X8 was used and study selection process documented using a PRISMA flow diagram [10].

### 2.2 Inclusion and exclusion criteria

Qualitative and quantitative studies reporting the views and experiences of key stakeholders in relation to implementation of self-consent procedures in vaccination programmes for young people aged between ten and 18 years were included [11]. Eligible stakeholders included young people, parents or primary caregivers, healthcare professionals, policy makers, community leaders and teachers. Studies related to consent procedures solely targeting parents of adolescents, or early childhood and adult vaccination programmes were not eligible. Studies which used interviews, focus groups, observations, surveys, and questionnaires with closed or open-ended questions allowing free-text responses were included. Conference abstracts, reviews, editorials, opinion pieces, dissertations, letters and books were included if they presented original data. No language or country of origin restriction was imposed.

### 2.3 Data extraction

Two reviewers (HF & SH) independently extracted data from selected studies using structured and standardised data extraction forms adapted from previous systematic reviews [6], [12]. Multiple publications related to the same study were reported together. Relevant domains such as study characteristics (e.g. authors, publication year, country), participant characteristics (e.g. participant age, sample size) and study results were retrieved. Any discrepancies were resolved through discussion.

### 2.4 Risk of bias

Assessment of risk of bias was undertaken independently by two reviewers (HF & SH) to illustrate potential sources of bias and recorded with an overall assessment of ‘low’, ‘moderate’, and ‘high’ in an excel spreadsheet. As the majority of eligible studies were observational, studies were not excluded on the basis of ‘high’ risk of bias if considered to contribute relevant information. We used the NIH Quality Assessment Tool for Observational Cohort and Cross-Sectional Studies [13] and the Critical Appraisal Skills Programme criteria adapted for qualitative studies [14]. For studies reporting both qualitative and quantitative data related to self-consent, the quality assessment tool related to the main data collection method used.

### 2.5 Data synthesis: Qualitative and quantitative studies

No single unifying framework exists for synthesising quantitative and qualitative evidence in systematic reviews [15]. The methodology for thematic synthesis reported by Thomas and Harden [16], which is suited to studies with a priori aims and objectives, was initially used to synthesise the qualitative data. The overall purpose of the qualitative synthesis was to ‘pool’ the results from individual primary studies by initially separating the findings, coding and interpreting the text, and then combining them through the identification of key themes across the studies as well as similarities and differences within those themes [17]. Thematic synthesis was carried out by one experienced reviewer (HF) who discussed interpretations of the data with a second reviewer (SA).

Familiarisation with the dataset began with reading the full papers. Pertinent sections of the text, including participant quotes and text written by the primary study authors, related to self-consent reported in the results section of each primary study represented the basic units for analysis and were uploaded to QSR NVivo11 software. An initial coding framework was developed, allowing for additional codes to emerge from the data. Refinements were made to the coding framework as data analysis progressed. During this process, overarching themes were identified, and differences or similarities explored within these emerging themes.

We originally planned to synthesise quantitative data through a meta-analysis, however this was not possible due to heterogeneity of outcomes and a lack of suitable data to calculate standardised effect sizes [18]. Initially, narrative descriptions of the results were created by extracting relevant key concepts and summarising findings [15]. Following consensus of the qualitative coding framework, the descriptions of individual quantitative studies were re-examined. As the focus of the qualitative and quantitative data were considered sufficiently similar, the quantitative data was subsequently integrated into a single synthesis based on the qualitative framework [19].

## 3 Results

**Fig. 2:**
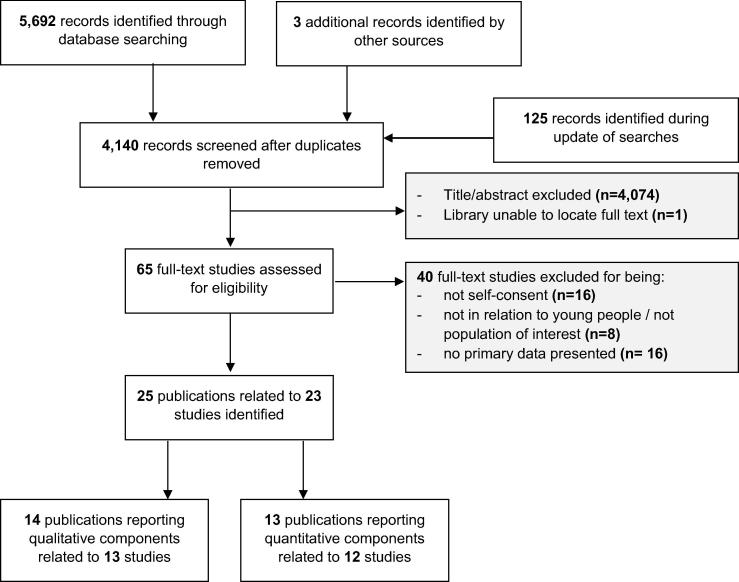
Flow diagram of study selection procedure.

Of 5,820 records identified, 4,140 abstracts were reviewed, and 65 full-text articles were assessed for eligibility. Full-text studies were excluded for: not being related to adolescent self-consent (n = 16); not being related to population group of interest (8), and; not presenting primary data (16). A total of 25 publications related to 23 studies met the inclusion criteria (Fig. 2).

The majority of studies were undertaken in UK (n = 10, 43.5%) and the USA (9, 39.1%), with one additional study each from Canada, Sweden, Australia and Tanzania. Most of the studies either used qualitative methodologies (11, 47.8%) or comprised a questionnaire (8, 34.8%). Additional single studies reported mixed methodologies, an educational intervention, a semi-qualitative questionnaire, and a needs assessment (methodology unclear). Twelve (52.2%) of the studies were in relation to HPV vaccination programmes, five (21.7%) referenced to multiple adolescent vaccination programmes, and the remaining six (26.1%) were non-specified adolescent vaccination programmes (Table 1).

**Table 1:** Description of primary studies.

Authors	Publi-cation year	Aim	Study Location -Geographical	Data collection period	Data collection methods	Sampling strategy	Analytical method	Participants	Vaccination programme	Authors conflicts of interest
Batista Ferrer, H, et al. [26], [27]	2015	To identify the barriers and facilitators to uptake of the HPV vaccine in an ethnically diverse group of young women in the south west of England	Three state-funded comprehensive schools in the south west of England, UK	Oct 2012-Jul 2013	Observations, semi-structured interviews & cross-sectional questionnaire	Purposive sample of schools and young women based on vaccine uptake, ethnicity, and Free School Meal entitlement	Thematic analysis & Framework approach to data management	Interviews: 6 key stakeholders & 23 ethnically diverse young women aged 12–13 years (68% response rate); questionnaire: 137 young women	HPV	Consultancy payment from GSK for a critical review of a health economic model of meningococcal ACWY vaccine
Brabin L, et al. [32]	2007	To explore parents' views on HPV vaccination in the context of adolescent autonomy	Eight schools in the city of Manchester, UK	Mar-Apr 2005	Semi-qualitative, cross-sectional questionnaire	A stratified sample of schools were invited based on school type and ethnicity; All parents of female year 7 pupils at participating schools	Descriptive analysis & summary of open-ended questions	317 parents of female students aged 11–12 years in participating schools (22% response rate)	HPV	Travel grants and funding from GSK for a vaccine clinical trial
Brabin L, et al. [34]	2009	To assess adolescent acceptance of HPV vaccination and the role of girls in the decision-making process	Two Primary Care Trusts, Manchester, UK	Not reported	Cross-sectional questionnaire	All daughters eligible for vaccination whose parents had agreed to participate in research	Fisher's exact tests	553 daughters aged 12–13 years (51%)	HPV	Travel grants and funding from GSK for a vaccine clinical trial
Braun R. [35]	2013	To assess HPV vaccination practices, use of the Vaccines for Children (VFC) program, and knowledge about recent legislation allowing for minor consent to HPV vaccination (AB499)	Health care organisations, California, USA	Not reported	Unclear (needs assessment)	Not reported	Not reported	44 Title X-funded health care organizations	HPV	Not reported
Brown E, et al. [23]	2010	To explore GPs’ and practice nurses’ views of HPV vaccination, prior to implementation of the national immunisation programme	Three general practices in Hampshire and Wiltshire, UK	Mar 2008	Semi-structured interviews	Convenience sample of general practices; recruitment of participants unclear	Thematic analysis & principles of constant comparison	10 general practitioners & 7 practice nurses	HPV	No conflicts of interest
Carolan K, et al. [36]	2018	To investigate the impact of two different education interventions on attitudes towards vaccination in young people	One secondary school in north west England, UK	Jan 2016 and Jul 2016	Pre- and post-intervention questionnaires (6-month follow-up)	Recruitment took place in one school: recruitment of participants unclear	Kruskal-Wallis & chi-square test	63 young people aged 14–15 years (94% White British)	Adolescent vaccines	Not reported
Ford C, et al. [24]	2009	To increase understanding of the policy, legal, and financial issues influencing efforts to achieve high rates of adolescent vaccination	Nine states, five jurisdictions, and nationally, USA	Not reported	Semi-structured telephone interviews	Purposive and snowballing	Thematic analysis	49 professionals with responsibilities for adolescent health care and/or vaccination (58% response rate)	Adolescent vaccines	Not reported
Ford C, et al. [37], [38]	2014	To explore whether, and to what extent, minor consent influences adolescent vaccine delivery in the United States	National sample of clinical settings	Feb-Apr 2009	Cross-sectional telephone questionnaire	Purposive sample of medical providers	Descriptive analysis	263 medical providers (49.4%) and public health professionals from state or jurisdiction immunization, STD, and family planning programs (50.6%) (72% response rate)	HPV, influenza & Tdap	Co-investigators on studies funded by GlaxoSmithKline
Gottvall M, et al. [25]	2015	To explore the relational aspects of the consent process for HPV vaccination as experienced by school nurses	Five towns/districts, Sweden	Apr-June 2010	Five focus groups	Towns/districts selected based on size & urbanicity; All eligible participants invited	Content analysis	30 school nurses (43% response rate)	HPV	Not reported
Hilton S, et al. [22]	2011	To investigate school nurses’ assessment of the HPV vaccine, their experiences of delivering the school based programme, and their views on parental decision-making about HPV vaccination	UK	Sept 2008- May 2009	Telephone interviews	Purposive sample based on experience, geographical location & location of schools	Constant comparative method	30 school nurses	HPV	No conflicts of interest
Humiston S, et al. [29]	2009	To assess health care providers’ attitudes and practices regarding adolescent immunizations, including factors that either impede or facilitate vaccination	Monroe County, New York & nationwide, USA	Spring 2005	Interviews & focus groups	Purposive sample based on location, practice type, setting & patient population	Grounded theory	Focus groups: 21 primary care practitioners (Monroe Country); Interviews: 24 key informant physicians & nurses (national)	Adolescent vaccines	Speakers’ bureaus and advisory boards for 3 companies that manufacture vaccines for adolescents
Kennedy A, et al. [39]	2012	To describe the vaccine-related knowledge and attitudes of adolescents aged 11–18 years and parents of adolescents aged 11–18 years	National, population-based, USA	2007	Cross-sectional questionnaire	A stratified random sample of 10,000 potential respondents from a panel of 600,000	Descriptive analysis	1,087 adolescents aged 11–18 years & 1,208 parents	HPV, MenACWY, &Tdap	Not reported
Kennedy C, et al. [30]	2014	To explore vaccination views in Scotland amongst parents, teenage girls and health professionals across three controversial vaccines	One health board in Scotland, UK	2008–2010	Interviews & focus groups	Purposive sample (methodology unclear)	Thematic analysis	51 healthcare professionals, 15 parents & 8 young women aged 12–15 years	HPV, influenza A, a& MMR	No conflicts of interest
Lee H, et al. [33]	2018	To identify potential barriers, facilitators and decision-making processes about HPV vaccinations among Hmong adolescents	One local community health centre, Minnesota, USA	Not reported	Focus groups	Not reported	Participatory thematic analysis	13 Hmong parents & 12 Hmong adolescents aged 14–17 years	HPV	Not reported
Marshall H, et al. [31]	2014	To seek adolescent and adult views on how existing adolescent school-based immunisation policy and program delivery could be improved to increase adolescent immunisation uptake	Metropolitan South Australia	2012	Citizen's juries	Stratified sampling to ensure demographically representative juries from a standing panel	Thematic analysis	15 adults & 16 adolescents aged 16–18 years	Adolescent vaccines	No conflicts of interest
Pyrzanowski J, et al. [40]	2013	To describe younger and older adolescents’ attitudes about health care and vaccination in five settings outside the traditional medical setting	Five schools in a large, urban public school district, USA	Apr-May 2008	Cross-sectional questionnaire	Purposive sample of schools based on ethnicity and socioeconomic diversity; all eligible students in participating schools	Descriptive analysis	392 adolescents aged 11–12 years (73% response rate); 296 adolescents aged 16–17 years (50% response rate)	Adolescent vaccines	No conflicts of interest
Rand C, et al. [41]	2011	To measure parent and adolescent perceptions about new adolescent vaccines	9 primary care practices, Monroe County, New York, USA	Mar 2007 -Apr 2008	Telephone cross-sectional questionnaire	Convenience sample	Chi-squared test	430 parents & 208 adolescents aged 15–17 years	Tdap, HPV, influenza & meningococcal	Not reported
Remes P, et al. [28]	2012	To learn what people knew about cervical cancer and HPV vaccination, whether they would find HPV vaccination acceptable, and how they viewed vaccine delivery and consent procedures	Two districts of Mwanza city & a neighbouring rural district (Misungwi), Tanzania	Mar-Aug 2010	Semi structured interviews & focus groups	Purposive	Thematic analysis	169 respondents (parents aged 18–59 years, religious leaders, teachers, health workers, female students aged 11–17 years)	HPV	Travel grants & funding from GlaxoSmithKline, Sanofi Pasteur MSD, Merck & Co. or Qiage
Rylance G, et al. [42]	1995	To determine children's views on consent issues	Two single sex schools in Birmingham, UK	Not reported	Cross-sectional questionnaire	Not reported	Descriptive analysis	513 students aged 11–15 years (60% response rate)	Measles & rubella	No conflicts of interest
Shah P, et al. [43]	2014	To examine the preferences for programmatic aspects of voluntary school mass vaccination programs	Population-based sample, USA	Nov 2011	Cross-sectional online survey	Participants from the HPV Immunization in Sons (HIS) longitudinal study	Paired t-tests	308 parents & 216 adolescent sons aged 11–19 years (78% follow-up)	Adolescent vaccines	Funding from Merck Sharp, Dohme Corp & GlaxoSmithKline
Stretch R, et al. [21]	2009	To assess school nurses views on assessing Gillick competence and vaccination of girls whose parents had not given consent	Two PCTs in Greater Manchester, UK	Not reported	Semi-structured interviews	All eligible school nurses delivering HPV vaccination programme	Thematic analysis	15 of 32 school nurses (46.9% response rate)	HPV	Travel grants & funding from GSK for a vaccine clinical trial
Wilson S, et al. [44]	2012	To evaluate the implementation of Ontario’s publicly-funded, school-based HPV immunization program through a process evaluation	36 Public Health Units, Ontario, Canada	Feb-Apr 2010	Cross-sectional questionnaire	Representatives at eligible Public Health Units	Descriptive analysis	41 vaccine-preventable disease managers at 36 Public Health Units	HPV	No conflicts of interest
Wood F, et al. [20]	2011	To explore the views of key stakeholders about how the process of consent should proceed where a potential conflict exists between parents	Wales, UK	Not reported	Semi-structured interviews	Convenience & snowballing	Thematic content analysis	25 professionals involved in the development of the HPV vaccination programme	HPV	No conflicts of interest

### 3.1 Qualitative studies summary

Fourteen publications [20], [21], [22], [23], [24], [25], [26], [27], [28], [29], [30], [31], [32], [33] relating to 13 studies reported relevant data using qualitative research methods within their study design. The most frequent data collection method was interviews [20], [21], [22], [23], [24], in addition to focus groups and interviews [28], [29], [30], interviews combined with observations [26], [27], focus groups [25], [33], semi-qualitative questionnaires [32], and citizen juries [31]. Study participants were diverse. Eleven of the studies comprised healthcare professionals [20], [21], [22], [23], [24], [25], [26], [27], [28], [29], [30], seven involved young people [30], [31], [33], [26], [27], [28], and five included parents [27], [28], [30], [32], [33] (Table 1).

### 3.2 Quantitative studies summary

Thirteen publications [27], [32], [34], [35], [36], [37], [38], [39], [40], [41], [42], [43], [44] relating to 12 studies reported relevant data using quantitative research methods. All used questionnaires to elicit responses from study participants. Participants comprised young people in eight of the studies [27], [34], [36], [39], [40], [41], [42], [43], parents of adolescents in four studies [32], [39], [41], [43], healthcare professionals in two studies [37], [38], and two studies were related to healthcare organisations [35], [44] (Table 1).

### 3.3 Risk of bias assessment

The majority of the studies reporting mainly qualitative data in relation to self-consent were considered at ‘low’ [25], [27], [20], [21], [22] or ‘moderate’ [23], [24], [28], [29], [30] risk of bias (Table 2). Three studies were considered at ‘high’ risk of bias [31], [32], [33]. All primary studies explicitly defined the aims of the research for which a qualitative research approach was appropriate. Only two studies presented a reflection on how the researcher could introduce bias during data collection or interpretation of the study findings [25], [27]. Study design increased the risk of bias being introduced to the study: one study classified as being at ‘high’ risk of bias incorporated a semi-qualitative research design using free text responses from a questionnaire study [32] and a second used ‘citizen juries’ [31]. For the third study at ‘high’ risk of bias, the data was presented as a poster with limited information available [33] (Table 2).

**Table 2:** Risk of bias for primary studies incorporating qualitative research methodologies.

Study author	*Clear statement of the aims of the research?*	*Qualitative methodology appropriate?*	*Research design justified?*	*Recruitment strategy appropriate?*	*Data collection methodology appropriate?*	*Relationship between researcher and participants considered?*	*Ethical issues been taken into consideration?*	*Data analysis sufficiently rigorous?*	*Clear statement of findings?*	*How valuable is the research?*	Overall risk of bias
Batista Ferrer H, et al. [26], [27]	✓	✓	✓	✓	✓	✓	✓	✓	✓	✓	Low
Brabin L, et al. [32]	✓	✗	✗	✓	✗	✗	✓	✓	✓	✗	High
Brown E, et al. [23]	✓	✓	✗	✗	✓	✗	✓	✓	✓	✓	Moderate
Ford C, et al. [24]	✓	✓	✗	✓	✗	✗	✓	✓	✓	✓	Moderate
Gottvall M, et al. [25]	✓	✓	✗	✓	✓	✓	✗	✓	✓	✓	Low
Hilton S, et al. [22]	✓	✓	✗	✓	✓	✗	✓	✓	✓	✓	Low
Humiston S, et al. [29]	✓	✓	✗	✓	✓	✗	✗	✓	✓	✗	Moderate
Kennedy C, et al. [30]	✓	✓	✗	✓	✓	✗	✓	✓	✓	✗	Moderate
Lee H, et al. [33]	✓	✓	✗	✗	✗	✗	✗	✗	✗	✗	High
Marshall H, et al. [31]	✓	✗	✓	✗	✓	✗	✓	✗	✓	✗	High
Remes P, et al. [28]	✓	✓	✗	✓	✓	✗	✗	✗	✓	✓	Moderate
Stretch R, et al. [21]	✓	✓	✗	✓	✓	✗	✓	✓	✓	✓	Low
Wood F, et al. [20]	✓	✓	✗	✓	✓	✗	✓	✓	✓	✓	Low

Of the studies reporting primarily quantitative data in relation to self-consent, all studies were classified as being at ‘high’ risk of bias [27], [32], [34], [35], [36], [37], [38], [39], [40], [41], [42], [43], [44], resulting from the use of descriptive, cross-sectional questionnaires that elicited subjective information from participants at a single time-point (Table 3).

**Table 3:** Risk of bias for primary studies incorporating quantitative research methodologies.

Study author	*Clearly stated research question?*	*Clearly defined study population?*	*Participation rate at least 50%?*	*Subjects recruited from similar populations?*	*Sample size justification?*	*Exposure of interest measured prior to outcome?*	*Timeframe sufficient between exposure and outcome?*	*Different levels of exposure measured?*	*Exposure(s) measures clearly defined?*	*Exposure(s) exposed more than once?*	*Outcome measures clearly defined?*	*Outcome assessors blinded to the exposure status?*	*Loss to follow-up less than 20%*	*Key confounders measured and adjusted for?*	Overall risk of bias
Batista Ferrer H, et al. [23], [24]	✓	✓	✓	✓	✗	✗	✗	**NA**	✗	✗	✗	**NA**	**NA**	✗	High
Brabin L, et al. [31]	✓	✓	✓	✓	✗	✗	✗	✓	✗	✗	✗	**NA**	**NA**	✗	High
Braun R. [32]	✓	✗	**NR**	**NR**	✗	✗	✗	**NA**	✗	✗	✗	**NA**	**NA**	**NR**	High
Carolan K, et al. [33]	✓	✓	**NR**	✓	✗	✗	✗	**NA**	✗	✗	✗	**NA**	**NA**	✗	High
Ford C, et al. [34], [35]	✓	✓	✗	✓	✗	✗	✗	**NA**	✗	✗	✗	**NA**	**NA**	✗	High
Kennedy A, et al. [36]	✓	✓	✓	✓	✗	✗	✗	**NA**	✗	✗	✗	**NA**	**NA**	✗	High
Pyrzanowski J, et al. [37]	✓	✓	✓	✓	✗	✗	✗	**NA**	✗	✗	✗	**NA**	**NA**	✗	High
Rand C, et al. [38]	✓	✓	**NR**	✓	✗	✗	✗	**NA**	✗	✗	✗	**NA**	**NA**	✗	High
Rylance G, et al. [39]	✓	✓	✓	✗	✗	✗	✗	**NA**	✗	✗	✗	**NA**	**NA**	✗	High
Shah P, et al. [40]	✓	✓	✓	✓	✗	✗	✗	**NA**	✗	✗	✗	**NA**	**NA**	✗	High
Wilson S, et al. [41]	✓	✓	✓	✓	✗	✗	✗	**NA**	✗	✗	✗	**NA**	**NA**	✗	High

NR: Not reported; NA: Not applicable

### 3.4 Themes

The data suggest that the implementation of adolescent self-consent procedures is influenced by three broad themes: the policy framework, notions of ‘protection’, and self-determination of young people. These are discussed below from the perspectives of the different stakeholders involved. Illustrative quotations were chosen because they were articulated concisely and typify responses relating to the themes.

### 3.5 Policy framework

Within the policy framework theme, issues related to implementation of self-consent procedures were grouped into the following sub-themes: national and local policy frameworks, understanding of legal guidelines, and context.

### 3.6 National and local policy frameworks

Seven studies undertaken in five countries discussed the national legal framework for assessing adolescent self-consent for vaccination. In the UK (England, Scotland and Wales) and Sweden, an adolescent who demonstrates themselves to be competent are legally entitled to provide consent for vaccination without their parents’ knowledge [20], [23], [25], [27], [30]. In the USA, young people are able to provide consent for healthcare that relates to sexually transmitted infections and family planning [24] and vaccinations conferring protection against sexually transmitted infections [35]. Despite the existence of a supportive national policy framework in the UK, local policy decisions may constrain healthcare professionals in assessing young women’s competency [20], [27]: “*From the nurse’s point of view, because we use a directive which says it must be parental written consent, we would not have been able to give it*” (Respondent 24, Wales) [20].

### 3.7 Understanding of legal guidelines

Some studies indicated lack of clarity regarding legal guidelines acted as a barrier to adolescent self-consent. Some professionals were aware of the legal framework for young people to consent to healthcare decisions, but appeared to disagree that it should be applied in the context of adolescent vaccination programmes [20], [23], [24]: “*I think it would be very unwise to simply say ‘This girl is Gillick competent, I’m not going to enter into discussion with the parents and I’m going to vaccinate her now’ ”* (Respondent 9, Wales, [20]). Often healthcare professionals incorrectly referenced national policy for obtaining young people’s consent [20], [21], [25], [27]. This included misperceptions of recent legislation changes in the USA which now allows adolescent self-consent for HPV vaccination (no further detail provided) [35], and reference to the UK Fraser guidelines which specifically address provision of contraceptive advice to young people without the knowledge of their parents, rather than vaccination [20], [21], [27]: “*We are using Fraser guidelines, which started off being on contraception and I know we can use it in all realms*” (School nurse 13, England, [21])*.* Similarly, parents were not always aware of the legal framework [32].

### 3.8 Context

Differing levels of acceptability of adolescent self-consent were evident by country and the setting of programme delivery. In the USA, data elicited from interviews indicated healthcare professionals felt obtaining consent from young people presenting for healthcare appointments unaccompanied was challenging [24], [29]: “*A whole bunch of adolescents will come in for preparticipation sports physicals. That is how they get their routine healthcare. We have not been giving immunizations. They do not routinely have parents present, and the issue of consent gets complicated*” (Key informant, USA, [24]).

In contrast, British and Swedish school nurses were generally more supportive towards self-consent for vaccinations provided it took place in a clinical context [20], [23], [25]. There was less support for self-consent to be assessed in the school setting. Barriers to implementation included concerns over school nurses’ competency [20] and practical aspects, such as time constraints and space, related to the delivery of the vaccination programme [20], [21]: “*If everybody is given 30 seconds to get in and out, you can’t reasonably expect a nurse to make a decision in that time. Yes or no? They would have to make some sort of later appointment to speak to the young person and have a serious chat, which in itself then would single them out from, you know if there was a mass queue*” (Respondent 17, Wales, [20]). School nurses indicated that they would routinely signpost young people who presented for vaccination without written parental consent to a clinical setting [25], [27]: “*I wouldn’t give them the shot myself, but I would give them a phone number and the name of someone they could go to*” (School nurse 3, Sweden, [25]).

Depending on the country, teachers could enable or constrain implementation of adolescent self-consent procedures. In Tanzania, some healthcare professionals were worried that teachers could coerce young people into attending vaccination sessions held at the school: “*When we go to administer a vaccine, we find the teachers have gathered the girls, and they are standing by the door with a stick*” (Health worker, Tanzania, [28]). Conversely, British teachers were less supportive of adolescent self-consent and were observed by a researcher in one study to physically prevent young people without written parental consent from entering the vaccination area [27]. No quantitative data was retrieved which related to this issue.

### 3.9 Protection

The views and actions of healthcare professionals, parents and young people in relation to adolescent self-consent for vaccination were shaped by different issues of ‘protection’. Whilst vaccinations may protect young people’s health, there were also concerns about protecting the reputation of professionals and their relationships with others, and the extent to which a young person’s confidentiality should be protected.

### 3.10 Young people’s health

Among participants there was acceptance that vaccines protect young people’s health [20], [21], [28], [32]. In some cases this related to higher perceptions of the risk of disease acquisition, which increased acceptability of self-consent procedures for parents and healthcare professionals [20], [23], [24], [32]: *“They should be able to request it (HPV vaccine) – if they are having sex and seem able to understand the issues*” (Parent, England, [32]). Lower perceptions of risk reduced acceptability [21]: *“I know the younger they have it the better, but there isn't the same degree of urgency* (for HPV) *and I would far rather sort it out and have everybody happy*” (School nurse 2, England, [21]). Conversely, self-consent procedures were considered to pose a clinical risk to young people: some parents and healthcare professionals believed that young people may not provide accurate medical history information [21], [32]: “*There could be a family history which may make it (the vaccine) unsuitable”* (Parent, England, [32]). No quantitative data was retrieved which related to this issue.

### 3.11 Reputation of professionals

The study based in Tanzania suggested that healthcare professionals were supportive of young people providing self-consent for vaccination, even when the parent had refused. This appeared to result from a community-wide acceptance that healthcare professionals act in the young person’s best interests: “*What I aim at is to save the life of the child, not the parent*” (Healthcare professional, Tanzania, [28]). In contrast, studies based in Sweden and the UK suggest the reputation of healthcare professionals and teachers could be jeopardised if parental preferences were not considered [20], [21], [23], [27], [45]: “*Because if you were to immunise a Gillick competent girl and her parents were dead against the vaccination, the ripples that that would cause in the school, you know ‘the nurse immunised my daughter even though we were against it’. It doesn’t really matter what the law would say, you’ve undermined confidence in the service. There are consequences*” (Respondent 9, Wales, [20]). Similarly, only a minority (39%) of representatives from Canadian healthcare organisations indicated they would consider vaccinating competent students in the school-setting in the absence of parental consent [44].

### 3.12 Relationships

Self-consent procedures could be enabled where the relationship between healthcare professional and young person was prioritised: “*Actually she’s being very responsible in coming and we want to encourage that. I still would feel extremely uneasy with going ahead with the immunisation although you begin to feel then that you are going against the fact that she is trying to do something very responsible*” (General practitioner, England, [23]). But more often, protecting relationships with parents was given precedence over implementation of self-consent procedures as healthcare professionals and teachers were fearful of repercussions [20], [21], [28]. Reaching consensus with the parent was preferred [20], [21], [23], [25]: “*Well, then we will have to do as we already do, and the parents will have to be present. Participate, simply enough*” (Nurse 4, Sweden, [25]). Similarly, parents emphasised their role in providing consent, suggesting that vaccination should not take place without their involvement [20], [30], [32]: “*I wouldn't like my daughter to make a lifechanging decision without being able to talk to me*” (Parent, England, [32]). Again, no quantitative data was retrieved which related to this issue.

### 3.13 Confidentiality

Concern over the protection of young people’s confidentiality varied. Following a vaccination request from a young person, healthcare professionals may routinely inform parents without regard for the young person’s right to privacy [20], [21]: “*I think you’d probably need to make some contact with the parents, just to make sure because I can understand that they would be perhaps rightfully a little bit irritated that they had never been told. Yeah, although the girl can consent on the day, I think the parents do need to be involved*” (Respondent 14, Wales, [20]). In the context of the UK HPV vaccination programme, some parents and healthcare professionals considered vaccination requests by young people could risk unintentional disclosure of sexual activity [23], [32]: “*If you’ve got a 14 year old girl whose consent is it that they have it? The theory being parental, but if they are sexually active and they haven’t told their parents, and their parents are saying ‘Yes I consent to her having it’, and she doesn’t want to say actually ‘There’s no point me having it because I’m already sexually active’ then they might end up having it when they might not want it or it might not be effective, and then they think they’re covered and they’re not*” (Practice nurse 16, England, [23]). This quotation also highlights a lack of understanding that a young person can still benefit from the vaccination if they are already sexually active. Some parents felt they should be informed if sexual behaviours were disclosed to healthcare professionals, with obvious implications for the confidentiality of young people [32]. No quantitative data was retrieved which related to this issue.

### 3.14 Self-determination

The self-determination theme encompassed issues related to parental responsibility, young people’s autonomy, and the characteristics of young people.

### 3.15 Parental responsibility

Young people’s self-determination to consent could be undermined by perceptions that the responsibility to provide consent for vaccinations belonged to the parent. In one study there was recognition that the consent process in the UK HPV vaccination programme favoured parents’ role in health-related decision-making for their children: “*I suppose the parents are going to override because they’ve got to actually sign the consent form*” (General practitioner 6, England, [23]).

Similarly, participants of qualitative studies felt strongly that parents should remain responsible for providing consent for adolescent vaccination [20], [21], [25], [27], [28], [30], [31], [32], [33]: “*Certainly with my younger daughter who was only 13 at the time…I would think well I ought to have been consulted on that*” (Mother, Scotland, [30]). Other adults suggested that where there was disagreement, parents’ vaccination preferences should be prioritised above that of their son or daughter [27], [28], [20], [21], [22]: “*If a child wants to have the vaccination, they need to talk to someone about having it done because their parents won’t let them have it and then interventions can be put into place. There is no way you can be giving a vaccination to a child without their parents’ consent. That is beyond crazy!*” (School teacher 3, England, [27]). This belief in parental consent might also be shared by young people. A questionnaire study in the USA showed that the majority of parents (70%) and their adolescent children (72%) believed that adolescents should not be able to self-consent for vaccination without parental knowledge and consent ahead of vaccination [39].

### 3.16 Young person’s autonomy

There was less evidence that participants supported young people to be autonomous and self-consent for vaccinations. A minority of British young people participating in an educational intervention (30.1%) believed at baseline that adolescents should have more say than their parents in relation to vaccination decisions [36]. However, some healthcare professionals and parents suggested that adolescents do have the right to make health-related decisions [20], [23], [27], [32], [33]: “*If they are old enough, regardless if you say no as parents, they have the right to go*” (Hmong Parent, USA, [33]). Participants in another study maintained that young people should be able to exercise autonomy and override parental refusal for vaccination: “*I should be vaccinated because I’m the one who’ll contract the disease*” (Student, Tanzania, [28]).

In a questionnaire study with British parents, 145 (48%) strongly agreed or agreed that young people should be able to consent for the HPV vaccine in a sexual health clinic without parental consent [32]. A small proportion (7%) of British young people offered the measles and rubella vaccine indicated they had been asked to consent to receive the vaccine. The majority did not believe that attending the immunisation session and cooperating with the vaccination process implied consent (65%) and believed that health professionals should not assume cooperation with the vaccination process implies consent (58%) [42].

### 3.17 Young person’s characteristics

Evidence from qualitative studies suggested that acceptability of self-consent procedures can be influenced by the perceived characteristics of the young person. The individual’s maturity and competency, as opposed to age, appeared to be the predominant factor for some parents and healthcare professionals [20], [21], [32], [33], [23], [24], [25]: “*You get some very mature but some look very tiny*” (School nurse 12, England, [21]). Young people were assumed as not capable of making the ‘right’ vaccination-related decision or avoiding vaccination due to fear of needles [30], [31], [32]: “*Children*… *will be informed but often times children are not the best judge to be able to weigh their decisions without parents*” (Parent, England, [32]).

The perceived acceptable age to self-consent varied by country. In the context of the Swedish and British HPV vaccination programmes, qualitative data highlighted that professionals and parents were reticent to implement self-consent procedures due to concerns that the target age for vaccination was too young (12–13 years old) [20], [21], [25], [32]. Healthcare professionals may be more willing to support self-consent procedures with older adolescents aged 15–16 years [21], [25]: “*If they are in high school and are 16 years old, then I think they should be able to decide, even if the parents say no. And then I would probably help them to get vaccinated without the parents’ consent*” (Nurse 3, Sweden, [25]).

In contrast, evidence from two questionnaire studies demonstrated that the majority (both 70%) of British young women aged 12–13 years believed they should be able to consent for vaccination without their parents’ consent [27], [34], with unvaccinated young women being less likely to agree [34]. In another study with young people, on average being aged 12.3 years was considered sufficient to demonstrate enough maturity and understanding to consent for vaccination [42]. More recently, around one-third (23/63) of young people aged 14–15 years agreed that well informed young people under the age of 16 should be able to receive vaccination without parental consent [36].

The perceived acceptable age for self-consent appeared to be older in the USA. In one study, 69% of parents and 40% of their adolescent sons believed that 18 years or older was an acceptable age for adolescents to provide consent for vaccination [41]. Another study showed that parents and sons believed on average 17 and 18 years, respectively, was an acceptable age for self-consent [43]. Differences by age were also evident in the USA: at least 54% of healthcare professionals would be willing to vaccinate a 17-year-old adolescent without parental consent, whereas only 34% would vaccinate a 12-year-old [37], [38]. Adolescents aged 16 to 17 years were reported to be more likely to agree that vaccination should take place without explicit parental consent than 11–12 years olds (74% vs. 30%, respectively) [40].

## 4 Discussion

### 4.1 Key findings

**Fig. 3:**
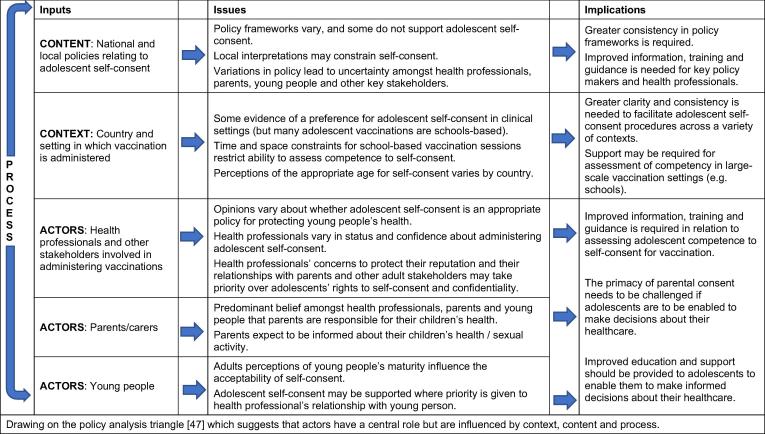
Summary of key issues from evidence synthesis and implications for practice.

This synthesis of barriers and enablers to the implementation of adolescent self-consent illustrates that local policies, professionals’ misunderstandings of the legal framework and the context in which the vaccination programme was delivered could prevent self-consent. Protection of young people’s health could act as an enabler to adolescent self-consent. However, preserving the reputation of professionals and existing relationships with parents acted as a major barrier and young people’s right to confidentiality was not always considered. Maintaining the role of parents as consenters for adolescent vaccinations was prioritised over enabling young people to be autonomous in decisions affecting their health. The target age for adolescent vaccination and perceptions of immaturity reduced the acceptability of adolescent self-consent procedures to adults and some young people. A summary of the key issues and implications for practice is summarised in Fig. 3.

### 4.2 Comparison to the wider literature

Allowing competent young people to self-consent has the potential to increase uptake of adolescent vaccination programmes. A recent systematic review showed complex, locally designed interventions (including social media campaigns, practice-based interventions, and reminders to parents) were most effective at increasing vaccine uptake and reducing inequalities [46]. As with the current systematic review, no intervention studies were captured that examined whether adolescent self-consent procedures could increase uptake, although one study is underway [47].

The methods for which legal frameworks for healthcare are developed can lead to uncertainty as to how the law should be practised in a clinical setting. Laws are evolved by judges in response to cases that appear in the court setting, and do not act as a comprehensive reference for healthcare professionals to follow [48]. Further, the function of adolescent consent can vary according to its application: transferring responsibility for decision-making is considered the objective of gaining legal consent, while the purpose of obtaining clinical consent is to facilitate treatment by fostering cooperation, enabling decision-making, preventing harm and doing good [48]. Further clarity is required at policy-level to ensure that the national legal framework in relation to adolescent consent is both applicable to, and practised within, local clinical frameworks.

A recent ethical discourse explored the issue of adolescent self-consent for HPV vaccination in the USA [49]. The authors concluded clinicians are ethically justified in vaccinating young people who make the request without parental consent. The authors argued self-consent enables the advancement of important public health goals with a negligible risk of harm through side-effects to the young person. Further, respecting young people’s right to make healthcare decisions could allow adolescents to develop autonomy and become responsible adults [49]. We consider these arguments should not be limited to vaccinations offering protection against sexually transmitted infections, as they are equally applicable to other adolescent vaccination programmes, and adolescent healthcare more generally.

### 4.3 Strengths and limitations

A systematic search of multiple databases was undertaken to identify all the relevant qualitative and quantitative literature meeting the predetermined study criteria. Studies were not excluded based on qualitative or quantitative research method, publication date, publication language, or population group. This has resulted in a comprehensive review capturing a range of perspectives in relation to barriers and enablers of self-consent for adolescent vaccination programmes. We used an integrative approach to synthesise qualitative and quantitative data [19] which allowed the possibility of reaching conclusions based on common elements identified across a range of studies. For example, few primary studies specifically addressed the protection of professionals’ reputation. However, when the studies were combined more data on this topic was revealed.

As the majority of the studies focused on the HPV vaccine, which protects against a sexually transmitted infection, it may be expected that issues related to self-consent may differ for other adolescent vaccinations. However, only three of the studies linked adolescent self-consent with the sexual transmission of HPV [23], [25], [32]. This suggests that the findings from this study are likely to be applicable to other adolescent vaccination programmes.

There are some limitations. First, and most critically, there were no intervention studies evaluating the impact of self-consent procedures on the uptake of adolescent vaccination programmes to synthesise, which had been the primary aim of our study. While only three of the qualitative studies included were at high risk of bias [31], [32], [33], all the eligible quantitative studies were considered at high risk. This was primarily due the choice of cross-sectional study design comprising subjective, questionnaire data. Therefore, the quantitative findings of this systematic review should be interpreted cautiously. It has not been possible to ascertain the prevalence of views related to some of the issues raised, such as confidentiality and relationships, as no quantitative literature was retrieved.

The findings from the primary studies did not explicitly consider the potential impact of self-consent procedures amongst different population groups, such as young people from socioeconomically disadvantaged backgrounds, minority ethnic groups, or who are considered more vulnerable such as those with an intellectual disability or looked after by the state and are already known to experience adverse outcomes across multiple health and social domains. Future studies should, where appropriate, incorporate experimental study designs, objectively measured outcomes, and be adequately powered to examine differences by different population groups.

Although no exclusion criteria based on study setting was applied, almost all studies meeting the inclusion criteria were from high-income countries. Therefore, the results from the study limits applicability of the findings within these settings. Further research to understand barriers and enablers to adolescent self-consent in low- and middle-income countries is suggested.

## 5 Conclusion

Our study findings show that implementation of adolescent self-consent procedures is governed by the policy context in which the vaccination programme is delivered and may be impeded by a desire to protect the reputation of professionals and the role of parents in decision-making. A key message from this synthesis is the need to clarify the policy framework in relation to adolescent vaccination programmes and challenge the primacy of parental consent. Supporting young people’s right to self-consent could help develop autonomy and encourage responsible decision-making in other health-related areas.

## Conflicts of interests

The authors declare there are no competing interests.

## Funding statement

This work presents independent research funded by the National Institute for Health Research (NIHR) under its Research for Patient Benefit (RfPB) Programme (Grant Reference Number PB-PG-0416-20013). The views expressed are those of the authors and not necessarily those of the NHS, the NIHR or the Department of Health. The work has been supported by the NIHR Health Protection Research Unit in Evaluation of Interventions at University of Bristol, in partnership with Public Health England (PHE). The work was also undertaken with the support of The Centre for the Development and Evaluation of Complex Interventions for Public Health Improvement (DECIPHer), a UKCRC Public Health Research Centre of Excellence. Joint funding (MR/KO232331/1) from the British Heart Foundation, Cancer Research UK, Economic and Social Research Council, Medical Research Council, the Welsh Government and the Wellcome Trust, under the auspices of the UK Clinical Research Collaboration, is gratefully acknowledged. The views and opinions expressed therein are those of the authors and do not necessarily reflect those of the NIHR RfPB Programme, the NHS, the Department of Health, or Public Health England.

## Authors contributions

The following contributions to the manuscript were made by the authors: concept and design of the study (HF, SA, JM and MH); reviewing titles and abstracts (HF and SH); data extraction (HF and SH); risk of bias assessment (HF and SH), and; analyses (HF and SA). HF wrote the first draft in collaboration with SA, and all authors contributed to the final version of the manuscript.
